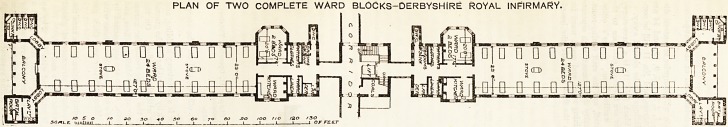# The New Derbyshire Royal Infirmary

**Published:** 1894-07-21

**Authors:** 


### The Institutional Workshop.

### HOSPITAL CONSTRUCTION.

### THE NEW DERBYSHIRE ROYAL INFIRMARY.

The story of the old infirmary at Derby and the causes which led to its final condemnation have been fully told in "Hospitals and Asylums of the World" (Vol. iv., p. 125). When it was finally decided to build a complete new hospital the governors endowed a large and representative committee with full powers to take the necessary steps of appointing an architect and preparing plans. A small sub-committee of this committee were charged with the duty of visiting the most recent hospitals in England, and from the knowledge thus gained, considered the question of recommending an architect who would be in their judgment best fitted to design and carry out a building in every respect up to the standard of modern knowledge. In the result, after having visited several hospitals and examined the plans of many others, the choice of the committee fell upon Messrs. Young and Hall, the architects of the Great Northern Central Hospital in London, the Hastings Hospital, and others; and they were accordingly instructed to prepare the plans for the buildings, the first portion of which are complete and were opened on Saturday week by the Duke of Devonshire.

During the preparation of the plans and, indeed, all through the time during which the buildings have been in progress the architects were in constant communication with the committee and the honorary medical staff, and the advantage that such intimate co-operation affords cannot be too highly appraised.

We give to-day a block plan of the whole of the buildings, with a detail plan of one ward pavilion, and in a future issue we propose to give a detail plan of the out-patient department.

The hospital when complete will comprise seventeen distinct blocks. Of these Nos. 1, 2, 3, 4, 5, 9, 13, and 14 are now complete; No. 6 is in progress, and No. 11 will be commenced as soon as the old buildings can be cleared away.

The front administration block (No. 1) is a three-storey building. On the ground floor are the main entrance to the hospital, secretary's office, waiting-room, porter's office, room for honorary medical staff, and the staff dining-room. At the east end is the casualty department, with separate entrance, waiting hall, examining-room, and small ward for cases of great urgency. On the first floor are the board-room and quarters for resident officers, and on the second floor the bed-rooms for servants.

1, Front Administration; 2, Kitchen and Stores; 3, Laundry; 4, 5, 6, 7, 8, Wards; 9, Out-patients; 10, Chapel; 11, Operation Theatre; 12, Eye Operation Room; 13, Nurses' Home; 14, Mortuary; 15, 16, Lodges; 17. Isolation.

NOTE.—The blocks shown in line only are not yet built. Area of site, 13 a. 0 r. 19 p.

The rear administration block is a two-storey building, and is connected with the front block by a corridor on the ground floor and by the flat roof over with the first floor. On the ground floor are the stores and the dining-rooms for nurses and servants. On the upper floor are the kitchen offices, and at the back the porters' bedrooms, which are approached by a separate staircase. The kitchens are lined throughout with glazed bricks, and fitted with Messrs. Slater and Co.'s gas and steam cooking apparatus.

From each side of the rear administration block runs the main corridor. This is a one-storey structure of glazed brick, with wood and glass superstructure and roof.

The laundry block (No. 3) is connected with No. 2 by an open covered way. It contains the general laundry and a separate small laundry for infectious linen. Here also is the boiler house, workshop, disinfecting apparatus, and cremator for refuse. Here also is a water-softening apparatus, through

PLAN OF TWO COMPLETE WARD BLOCKS—DERBYSHIRE ROYAL INFIRMARY.

which the whole of the water is passed before being distributed to the various points.

The ward pavilions (Nos. 4 and 5) are of two storeys, the lower storey being in each case well raised above the ground on open arches; an arrangement which permits of free circulation of air underneath the block and avoids the stagnation of air in the angle formed by the ground and the upright walls. The importance of this precaution, so well recognised abroad, has seldom been considered in this country; the only two instances we can recall being the small isolation block built some years ago at the London Fever Hospital and the recently erected wing at the Warneford Hospital, Leamington.

The two pavilions are divided by the main corridor, on one side of which is the staircase; the connection between the staircase and the ward pavilion in one case, and the corridor and the ward pavilion in the other, is by means of a covered bridge, cross-ventilated, and provided with swing doors at each end; these bridges are of a height sufficient for headway only, and the space between the roof of one and the floor of that above is entirely open.

At the entrance to each pavilion on each floor will be found the various ward offices; a food store, broom cupboard, linen store, store for patients' clothes, ward kitchen, and a w.c. for the use of nurses; there is also a separation ward for two beds. The large wards are 127 ft. long, 29 ft. wide, and 14 ft. 3 in. high, and are intended for 24 beds each. The allowance of floor space is at the rate of 145 sq. ft. per bed, and of cubic space 2,066·25 ft. per bed. Each bed is placed between two windows, and there are no beds in the angles. The surfaces of the walls and ceilings are of Keen's cement, painted and varnished. The windows are fitted with double hung sashes with a hopper-light above the transom, the sides of which are protected by glazed spandrils. The bath-rooms and water-closets are placed at the further end of the wards in projecting towers, approached by cross-ventilated covered bridges. Between these towers are wide balconies for the use of convalescent patients.

The floors and ceilings are constructed of iron girders and joists encased in concrete. The floors are finished with terrazzo, a mixture of Portland cement and marble chips rubbed smooth and polished. The question of the most suitable surface for the ward floors was not settled without careful and anxious consideration. It was at first intended to use oak or teak blocks bedded on the concrete surface in mastic; but it was found that however well such a floor may be laid, and however well seasoned the wood may be, the blocks are liable to shrink in the neighbourhood of fire-places or hot-pipes, or to be tilted up by the pressure of the legs of beds or other heavy pieces of furniture. To these defects a terrazzo floor is not liable; it is, on the other hand, absolutely impervious. When properly laid it has no joints; it is not so slippery as a polished wooden floor, and consequently not so tiring to walk on; it is easily cleaned, and soon dries. The only objection that is seriously made is on the score of its alleged coldness; but inasmuch as in Hamburg, where the climatic conditions are said to be almost exactly similar to those of England, and in Berlin, where the winter is usually more severe than with us, these floors have been in use for many years, and no inconvenience on this head is experienced, this objection may fairly be disregarded.

In the finishing of all the joiners' work and at the junction between the walls and ceilings and floors all the angles are rounded to prevent lodgment of dust and to promote free circulation of air.

The wards are provided with two sets of double grates with descending flues, the grates being formed on the Pridgin-Teale system, and cased externally with glazed faience ware by the Burmantofts Company. In addition to the grates there are in each ward ten steam coils set in recesses under the windows, and enclosed in iron cases. At the back of each coil is a channel in the thickness of the wall for the admission of fresh air, which is controlled by a valve worked from inside the ward by a lever. The warmed air enters the ward through an opening in the top of the casing, and the amount to be admitted can be controlled at will. The front of the coil-case is hinged in two leaves, so that it can be opened to get at the coil; the latter was specially designed by Messrs. Slater and Co. in conjunction with the architects. Each coil is formed of copper tubes in three leaves, each leaf being hung on steam-tight hinges, so that it can be swung out for the purpose of cleaning, and each leaf can be used independently of the other two.

In ward block (No. 6), which, as already stated, is in progress, the upper floor will be devoted partly to the gynæcological department and partly to sick nurses. It will contain a general ward for ten beds, one ward for two beds, and four wards for one bed each, with an operation-room and the usual ward offices.

The operation block (No. 11) will be a one storey building and will contain the operation-room, a room for the administration of anæsthetics and a room for the staff. The operation-room will be lined to a height of seven feet with marble slabs, above which the walls and ceiling will be finished with Keene's cement painted and varnished. The floor will be laid with marble mosaic; and the whole room, with its fittings, will be so arranged that it can be swilled out with a hosepipe.

The nurses' home (No. 13) has been in occupation for some months. It is entirely detached, is of three storeys in height, and affords accommodation for 48 nurses. Each nurse has a separate bedroom and separate sitting-rooms, and readingrooms have been provided for the sisters, staff nurses, and probationers respectively.

The mortuary block (No. 14) is also a detached building, and contains a dead-house and post-mortem room, and a mortuary chamber lined with glazed tiles, in which a body can be placed for friends to view; attached to this block are a stable and ambulance-house.

The chapel (No. 10) is to be erected as a memorial to the late Sir William Evans, the president of the infirmary when the foundation stone was laid by Her Majesty the Queen. The plans are still under discussion.

The eye and children's block (No. 8) with the eye operation-room (No. 12), are deferred for the present for want of funds, and a temporary structure will be erected shortly for the accommodation of the eye department. Ward block No. 7, he "future extension" block, will probably not be required for some years to come.

The drainage is separated into two distinct systems; one for rain-water from the roof, which is conducted to a large storage tank, from whence it is pumped for use in the laundry, &c., and the other for the soil drainage and surface drainage from the roads, &c., which is connected with the town sewers. The drains are all laid out in straight lines from point to point, with manholes at all junctions or changes of direction, and at the head of each main line of drain is a flushing tank fitted with a Field's automatic syphon.

The contractors for the building are Messrs. Walker and Slater, of Derby; the internal plumbing has been carried out by Messrs. Dent and Hellyer; the steam-heating, hot-water work, and cooking apparatus, by Messrs. Slater; the laundry fittings by Messrs. Bradford; the electric bells and telephones by Messrs. Davis; electric lighting by Messrs. Haslam; and the formation of roads and laying-out of the grounds by Messrs. Barron.

## Figures and Tables

**Figure f1:**
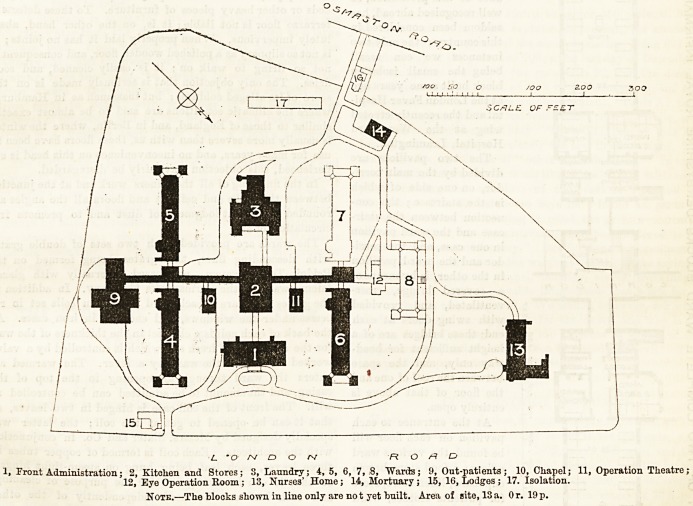


**Figure f2:**